# Incidence and Patterns of Interstitial Lung Disease and Their Clinical Impact on Mortality in Patients with Antineutrophil Cytoplasmic Antibody-Associated Vasculitis: Korean Single-Centre Observational Study

**DOI:** 10.1155/2022/2499404

**Published:** 2022-05-23

**Authors:** Jang Woo Ha, Jung Yoon Pyo, Sung Soo Ahn, Jason Jungsik Song, Yong-Beom Park, Sang-Won Lee

**Affiliations:** ^1^Division of Rheumatology, Department of Internal Medicine, Yonsei University College of Medicine, Seoul, Republic of Korea; ^2^Institute for Immunology and Immunological Diseases, Yonsei University College of Medicine, Seoul, Republic of Korea

## Abstract

**Objectives:**

This study investigated the frequency and patterns of interstitial lung disease (ILD) and their clinical effect on all-cause mortality during the follow-up period in patients with antineutrophil cytoplasmic antibody- (ANCA-) associated vasculitis (AAV) in Korea.

**Methods:**

The medical records of 255 AAV patients with ILD were retrospectively reviewed. ILD and its patterns, the usual interstitial pneumonia (UIP) and non-UIP patterns, were confirmed using high-resolution computed tomography both at AAV diagnosis and during follow-up. Forced vital capacity (FVC) and diffusing capacity of the lung for carbon monoxide (DLCO) were also obtained.

**Results:**

The median age was 65.0 years, and 34.9% were male. ILD occurred in 53 patients, among whom 49.1% developed ILD after AAV diagnosis. Among AAV subtypes, the frequencies of ILD were significantly higher in both patients with microscopic polyangiitis (MPA) and those with AAV having myeloperoxidase (MPO)-ANCA (or P-ANCA) compared to other subtypes. However, there was no statistical significance in AAV subtypes or FVC/DLCO ratio between patients with the UIP and non-UIP patterns. In particular, the cumulative patients' survival rate was lower in patients with AAV and ILD than in those without ILD.

**Conclusions:**

ILD occurred in one-fifth of Korean patients with AAV in this study and was associated with MPA and MPO-ANCA (or P-ANCA). In addition, ILD significantly increased the rate of all-cause mortality in these patients with AAV. Therefore, we suggest the need for more attention and more frequent regular visit for patients with AAV and ILD regardless of the time of ILD occurrence.

## 1. Introduction

Antineutrophil cytoplasmic antibody- (ANCA-) associated vasculitis (AAV) is a necrotising vasculitis that affects small-sized vessels, mainly capillaries, venules, and arterioles and occasionally adjacent arteries [1]. Since AAV can invade almost all major organs, it may exhibit various clinical manifestations and is generally classified into microscopic polyangiitis (MPA), eosinophilic granulomatosis with polyangiitis (EGPA), and granulomatosis with polyangiitis (GPA) based on clinical, laboratory, radiological, and histological features [1–3]. Furthermore, the name, “AAV,” was derived from autoantibodies, MPO-ANCA, and PR3-ANCA, which recognise neutrophil cytoplasmic autoantigens such as MPO and PR3 and play an important role in the pathogenesis of AAV [4]. Therefore, AAV is also classified into MPO-ANCA vasculitis, PR3-ANCA vasculitis, and ANCA-negative vasculitis based on the presence of ANCA types [5].

Among nine clinical categories based on version 3 of the Birmingham vasculitis activity score (BVAS) version 3, a pulmonary manifestation of AAV includes wheezing, nodules or cavities, pleural effusion, pleurisy, infiltrate, endobronchial involvement, massive haemoptysis/alveolar haemorrhages, and respiratory failure. Of these, some cases of “pulmonary infiltrate” may be associated with interstitial lung disease (ILD) [6]. ILD is a group of diffuse parenchymal lung disorders and is divided into two patterns, namely, usual interstitial pneumonia (UIP) and non-UIP, using high-resolution computed tomography (HRCT) [7].

Previous meta-analyses have reported the clinical implications of ILD in patients with AAV: (i) ILD occurs in approximately 23% of patients with GPA and up to 45% of those with MPA; (ii) ANCA positivity might be associated with the concurrent occurrence of ILD in patients with AAV: in particular, MPO-ANCA might be detected in 46-71% of patients with AAV and ILD; (iii) the UIP pattern is predominantly found in patients with AAV ranging from 43% to 83%; and (iv) ILD is likely associated with a relatively high mortality rate in patients with AAV, in particular, those with the UIP pattern [8, 9]. A previous study reported that 24 of 74 (32.4%) Korean patients with AAV having both HRCT and histological results presented the ILD patterns, and 13 of 24 (54.2%) showed the UIP pattern. Furthermore, it is demonstrated that lung biopsy was useful in resolving discordance between radiological and histological findings in patients with AAV [10].

However, to date, no studies have reported the frequency and patterns of ILD and their clinical effect on all-cause mortality during the follow-up period in Korean patients with AAV. Hence, this study included only patients with AAV and ILD confirmed using HRCT both at AAV diagnosis and during follow-up and investigated the frequency and patterns of ILD and their clinical effect on all-cause mortality during the follow-up period in patients with AAV in Korea.

## 2. Materials and Methods

### 2.1. Study Subjects

The medical records of 255 patients with AAV enrolled in the Severance Hospital ANCA-associated VasculitidEs (SHAVE) cohort were retrospectively reviewed. The SHAVE cohort is an observational cohort of patients with AAV of a single centre. As described in the previous study [11, 12], the inclusion criteria were patients (i) classified as having MPA, EGPA, or GPA at the Division of Rheumatology, Department of Internal Medicine, Yonsei University College of Medicine, Severance Hospital between May 2000 and June 2021; (ii) who fulfilled both the 2012 revised International Chapel Hill Consensus Conference Nomenclature of Vasculitides and the 2007 European Medicine Agency algorithm for the classification of ANCA-associated vasculitides [1, 2]; (iii) who had the medical records with sufficient data on clinical, laboratory, radiological, and histological data for the classification of AAV and the occurrence of ILD and all-cause mortality; (iv) for whom HRCT was performed when ILD was suspected; (v) who had been followed up for at least 3 months; (vi) who had no other diseases with a clear medical association as the cause of ILD except for AAV; (vii) who had no concomitant medical conditions mimicking AAV as well as ILD such as serious infectious diseases, malignancies, and systemic vasculitides other than AAV; and (viii) who had never been exposed to glucocorticoids equivalent to prednisolone > 20 mg/day before AAV diagnosis. Coexisting serious medical conditions and immunosuppressive drug use were identified using the 10th revised International Classification of Diseases and the Korean Drug Utilization Review system, respectively. This study was approved by the Institutional Review Board (IRB) of Severance Hospital (Seoul, Korea, IRB No. 4-2021-1562) and was conducted according to the principles of the Declaration of Helsinki. Given the retrospective design of the study and the use of anonymised patient data, the requirement for written informed consent was waived.

### 2.2. Clinical Data

Demographic data, AAV-related variables, and clinical data at diagnosis were collected and are shown in Table 1. Diabetes mellitus, hypertension, and dyslipidaemia were evaluated as comorbidities at AAV diagnosis. The follow-up period based on ILD was defined as the period from AAV to ILD diagnosis. All-cause mortality was defined as death owing to any cause regardless of its association with the disease entity of AAV. The follow-up period based on all-cause mortality was defined as the period between AAV diagnosis and the last visit for surviving patients and as that between AAV diagnosis and the death as for deceased patients. The follow-up period from ILD diagnosis to mortality was defined as the period between ILD diagnosis and the last visit for surviving patients and as the period between ILD diagnosis and death for deceased patients [11].

### 2.3. ILD Patterns and Pulmonary Function Test

This study only considered ILD confirmed using HRCT. The UIP and non-UIP patterns were confirmed by a radiologist specialising in lung imaging. Within 2 weeks of HRCT performance, pulmonary function tests (PFTs) were performed in 45 patients with AAV and included forced vital capacity (FVC) and diffusing capacity of the lung for carbon monoxide (DLCO).

### 2.4. Statistical Analyses

All statistical analyses were performed using IBM SPSS Statistics for Windows, version 26.0 (IBM Corp., Armonk, NY, USA). Continuous and categorical variables are expressed as medians with interquartile ranges and numbers (percentages), respectively. Significant differences between categorical variables were analysed using chi-square and Fisher's exact tests. The Mann-Whitney *U* tests were used to compare significant differences between two continuous variables. Comparison of the cumulative survival rates between the two groups was performed using the Kaplan-Meier survival analysis with log-rank tests. Statistical significance was set as *P* < 0.05 [11].

## 3. Results

### 3.1. Baseline Characteristics

At AAV diagnosis, the median age was 65.0 years, and 34.9% of patients were male. Among 255 patients with AAV, 138 were classified as having MPA, 52 as having EGPA, and 62 as having GPA. ANCA positivity was reported in 80% of patients with AAV. The medial BVAS and FFS were 12.0 and 1.0. The most common clinical manifestations were renal and pulmonary conditions (60.8% for each). ILD occurred in 53 patients with AAV (20.8%) during a median follow-up period based on ILD of 0 months (an interquartile range of 4.1). Among 53 patients with AAV and ILD, ILD was found at AAV diagnosis in 27 patients (50.9%) and after AAV diagnosis in 26 patients. Furthermore, UIP was identified in 29 patients with AAV and ILD (54.7%). In addition, 32 patients with AAV (12.5%) died owing to any cause during a median follow-up period based on all-cause mortality of 37.2 months (49.1%). The median follow-up period from ILD diagnosis to mortality was 40.5 months.

### 3.2. Comparison of ILD Patterns among Three AAV Subtypes

**Figure 1:**
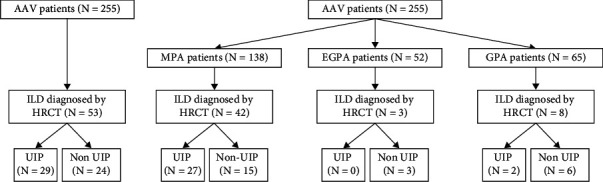
Algorithm for subject selection. AAV: ANCA-associated vasculitis; ANCA: antineutrophil cytoplasmic antibody; MPA: microscopic polyangiitis; EGPA: eosinophilic granulomatosis with polyangiitis; GPA: granulomatosis with polyangiitis; ILD: interstitial lung disease; HRCT: high-resolution computed tomography; UIP: usual interstitial pneumonia.

ILD was identified in 42 patients with MPA (30.4%), 3 patients with EGPA (5.8%), and 8 with GPA (12.3%). Furthermore, the UIP pattern was confirmed in 27 of 42 patients with MPA and ILD (64.3%) and in 2 of 8 GPA patients with ILD (25.0%). However, no patients with EGPA and ILD turned out to have the UIP pattern (Figure 1). Patients with MPA had significantly higher frequencies of ILD than those with EGPA and GPA (*P* < 0.001 and *P* = 0.005); however, there was no significant difference between patients with EGPA and those with GPA. Patients with MPA tended to have higher frequencies of the UIP pattern than those with EGPA and GPA, but the difference was not statistically significant (Table 2).

### 3.3. Comparison of ILD Patterns according to ANCA Type

The frequency of ILD was significantly higher in patients with AAV having MPO-ANCA (or P-ANCA) than in those not having MPO-ANCA (or P-ANCA) (28.2% vs. 5.9%, *P* < 0.001). Conversely, the frequency of ILD was significantly higher in patients with AAV not having PR3-ANCA (or C-ANCA) than in those having PR3-ANCA (or C-ANCA) (24.2% vs. 4.6%, *P* = 0.004). However, the frequencies of the UIP patterns did not differ significantly between the two groups based on ANCA type (Table 3).

### 3.4. Comparison of Pulmonary Function Tests according to ILD Patterns

Among 53 patients with AAV and ILD, 45 underwent PFTs within 2 weeks of HRCT performance. Comparisons of patients with and without the UIP pattern showed no significant differences in FVC or DLCO (Table 4).

### 3.5. Comparison of Cumulative Patients' Survival Rates

**Figure 2:**
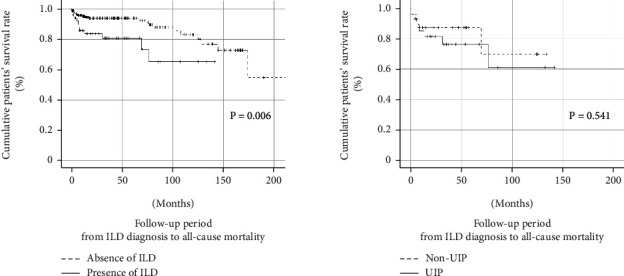
Comparison of cumulative patients' survival rates in AAV patients. ILD: interstitial lung disease; UIP: usual pneumonia; AAV: ANCA-associated vasculitis; ANCA: antineutrophil cytoplasmic antibody.

Among 255 patients with AAV, the cumulative patients' survival rate was lower in those with ILD than in those without ILD (*P* = 0.006). However, 53 AAV patients with ILD showed no difference in the cumulative patients' survival rates between those with and without the UIP pattern (Figure 2).

Patients with AAV and ILD exhibited a significantly lower cumulative patients' survival rate than those without ILD. However, the rates did not differ between patients with AAV with and without the UIP pattern.

## 4. Discussion

This study investigated ILD patterns and their clinical effect on all-cause mortality in Korean patients with AAV and obtained several clinical findings. First, ILD occurred during follow-up in 53 patients with AAV (20.8%), among whom 49.1% developed ILD after AAV diagnosis. Second, among the AAV subtypes, ILD occurred more frequently in patients with MPA than in those with EGPA and GPA. Furthermore, among ILD patterns, the UIP pattern tended to be observed more often in patients with MPA than in those with other subtypes; however, the difference was not statistically significant. Third, patients with AAV having MPO-ANCA (or P-ANCA) showed significantly higher frequencies of ILD compared to those not having MPO-ANCA (or P-ANCA). Conversely, patients with AAV having PR3-ANCA (or C-ANCA) less frequently had ILD compared to those not having PR3-ANCA (or C-ANCA). Finally, during the follow-up period, patients with AAV and ILD exhibited a significantly lower cumulative patients' survival rate than those without ILD but were not applicable to the UIP pattern.

In the present study, the frequency of ILD in patients with AAV was 20.8% (Table 1), which was higher than that in patients in London (2.7%) but lower than that in Japanese patients (39.1%) [13]. This discrepancy suggests that the frequency of ILD depends on ethnic and geographic features. However, the frequencies of ILD in patients with MPA and GPA were found to be 30.4% and 12.3%, respectively (Table 2), which were remarkably low rates compared to those reported previously [9]. One explanation for the relatively low frequency of ILD in patients with MPA and GPA was the short follow-up period. It is expected that a longer follow-up period would likely show a proportion of patients with ILD after AAV diagnosis above 49.1%. The higher occurrence rate of ILD among MPO-ANCA-positive patients than that in PR3-ANCA-positive patients was consistent with the findings of previous studies, which also support the effect of the short follow-up period on the low frequency of ILD in this study [14]. The 2022 classification criteria for MPA recently proposed by the American College of Rheumatology and the European Alliance of Associations for Rheumatology assigned the highest score of +6 to the item of “MPO-ANCA positivity” and a score of +3 to a newly added item of “fibrosis or ILD on chest imaging.” It can be said that the results of the present study are in the same context as the background in which these scores are assigned in the new criteria for MPA [15].

Previous studies showed that the UIP pattern is more likely to be associated with all-cause mortality than the non-UIP pattern in patients with AAV and ILD. A meta-analysis reported that patients with AAV and the UIP pattern had a higher risk of poor prognosis, including all-cause mortality (RR 4.36) [8]. However, the present study showed no significant difference in the cumulative patients' survival rates between patients with and without the UIP pattern among 53 patients with AAV and ILD.

We propose explanations for this discrepancy. The first is the short follow-up period. Although approximately half of the patients developed ILD after AAV diagnosis, the median follow-up period based on ILD was 0 months, which supports this hypothesis. Future studies are needed to assess the difference in the survival rates between patients with and without the UIP pattern in the longer follow-up periods.

The second explanation is the relationship between ILD patterns and AAV subtypes. Patients with MPA tended to present with the UIP pattern more frequently than patients with EGPA or GPA (*P* = 0.058 and *P* = 0.056, respectively). Among 32 deceased patients, 20 of 138 patients with MPA (14.5%), 1 of 52 patients with EGPA, and 11 of 65 patients with GPA (16.9%) died. A comparison of patients with MPA and GPA showed that although the proportion of the non-UIP pattern was higher in patients with GPA, the overall mortality was comparable to that of MPA.

The third explanation is the similar FVC and DLCO between the two groups. A previous study reported an increased mortality rate with an increasing FVC/DLCO ratio because a decrease in DLCO was more closely related to an increase in all-cause mortality through the development of pulmonary hypertension [14]. However, in this study, the differences were not statistically significant: patients with AAV without the UIP pattern had a lower median DLCO than patients with the UIP pattern (68.0% vs. 74.0%), and the FVC/DLCO ratio was slightly higher in patients with AAV without the UIP pattern than in those without (1.25 vs. 1.11). These hypothetical factors may offset the increase in all-cause mortality owing to the UIP pattern in patients with AAV and ILD.

In this study, no EGPA patients had the UIP pattern. According to the classification criteria for EGPA, a typical lung lesion is defined as a nonfixed (migratory) lung infiltrate [1, 2]. In real clinical settings, the definite UIP pattern might have contributed to the classification of MPA or GPA rather than EGPA in patients suspected of AAV. In other words, it was not that EGPA patients can never show the UIP pattern, but that patients with the UIP pattern did not meet one criterion for the classification criteria for EGPA. There were few comprehensive previous studies on the UIP pattern in a considerable number of EGPA patients to date.

When only 26 patients with AAV, who had ILD after AAV diagnosis (identical to during follow-up), were considered as AAV patients with ILD, the significant differences in the incidence of ILD between MPA and EGPA patients and that between MPA and GPA patients, which were observed in Table 2, disappeared (Supplementary Table 1). In addition, the incidence of ILD still exhibited a significant difference between patients with MPO-ANCA (or P-ANCA) and those without; however, it was not found between those with and without PR3-ANCA (or C-ANCA) (Supplementary Table 2). However, like Table 4, there were no significant differences in both FVC and DLCO between patients with UIP and those with non-UIP (Supplementary Table 3). It could be concluded that MPO-ANCA (or P-ANCA), but not AAV type, might contribute to the incidence of ILD in AAV patients during follow-up, and there were still no differences in lung functions regardless of UIP or non-UIP. Therefore, closer attention regarding ILD occurrence by regularly performing chest imaging in AAV patients having MPO-ANCA (or P-ANCA) at AAV diagnosis [16].

On the other hand, the cumulative patients' survival rates according to the presence of ILD or UIP patterns in 26 patients who had ILD after the diagnosis of AAV were compared between the two groups. Patients with ILD tended to exhibit a lower cumulative patient's survival rate than those without ILD; however, no statistical significance was observed, whereas there was no significant difference between patients with UIP and those with non-UIP (Supplementary Fig. 1). Therefore, it might be concluded that not only ILD occurrence at or before AAV diagnosis but also ILD development after AAV diagnosis contributed to all-cause mortality in AAV patients together.

The strength of this study is that it is the first to investigate the frequency of ILD and the effects of ILD and its patterns on all-cause mortality in patients with AAV in Korea. This study also had some limitations. First, we could not determine the exact time of ILD occurrence in patients with AAV who had ILD at AAV diagnosis. Patients with AAV who might have had ILD before AAV diagnosis were considered to have ILD at AAV diagnosis. As this may alter the follow-up duration from ILD diagnosis to all-cause mortality, it may have influenced mortality outcomes according to the UIP or non-UIP patterns. Only 45 of 53 AAV patients with ILD underwent PFTs, which may have confounded the analysis of the correlation between PFT results and the mortality rate. Additional limitations include the small number of patients with AAV due to the single centre and retrospective study. A future prospective study with a larger number of patients and serious HRCT and PFT results will provide more dynamic and reliable data on the clinical implications of ILD on the prognosis of patients with AAV.

## 5. Conclusion

ILD occurred in one-fifth of Korean patients with AAV in this study and was associated with MPA and MPO-ANCA vasculitis. In addition, ILD significantly increased the rate of all-cause mortality in these patients with AAV. Therefore, we suggest the need for more attention and more frequent regular visit for patients with AAV and ILD regardless of the time of ILD occurrence.

## Figures and Tables

**Table 1:** Baseline characteristics of patients with AAV at diagnosis (*N* = 255).

Variables	Values
Demographic data at AAV diagnosis	
Age (years)	65.0 (14.0)
Male gender (*N* (%))	89 (34.9)
Ex-smoker (*N* (%)	9 (3.5)
Body mass index (kg/m^2^)	22.4 (4.13)
AAV subtype at AAV diagnosis (*N* (%))	
MPA	138 (54.1)
EGPA	52 (20.4)
GPA	65 (25.5)
ANCA positivity at AAV diagnosis (*N* (%))	
MPO-ANCA (or P-ANCA) positivity	170 (66.7)
PR3-ANCA (or C-ANCA) positivity	44 (17.3)
Both ANCA positivity	10 (3.9)
ANCA negativity	51 (20.0)
AAV-specific indices at AAV diagnosis	
BVAS	12.0 (11.0)
FFS	1.0 (1.0)
Clinical manifestations at AAV diagnosis (*N* (%))	
General	108 (42.4)
Cutaneous	55 (21.6)
Mucous membranous/ocular	14 (5.5)
Otorhinolaryngological	118 (46.3)
Pulmonary	155 (60.8)
Cardiovascular	55 (21.6)
Gastrointestinal	12 (4.7)
Renal	155 (60.8)
Nervous systemic	84 (32.9)
Comorbidities at AAV diagnosis	
Diabetes mellitus	65 (25.5)
Hypertension	101 (39.6)
Dyslipidaemia	47 (18.4)
Glucocorticoids and immunosuppressive drugs during follow-up	
Glucocorticoids	239 (93.7)
Cyclophosphamide	138 (54.1)
Rituximab	41 (16.1)
Mycophenolate mofetil	37 (14.5)
Azathioprine	135 (52.9)
Tacrolimus	22 (8.6)
Methotrexate	24 (9.4)
ILD during follow-up	
ILD (*N* (%))	53 (20.8)
ILD at (or before) AAV diagnosis	27/53 (50.9)
ILD after AAV diagnosis	26/53 (49.1)
UIP (*N* (%))	29/53 (54.7%)
Non-UIP (*N* (%))	24/53 (45.3%)
Follow-up period based on ILD (months)	0 (4.1)
All-cause mortality during follow-up	
All-cause mortality (*N* (%))	32 (12.5)
Follow-up period based on all-cause mortality (months)	37.2 (66.2)
Follow-up period from ILD diagnosis to mortality (months)	40.5 (65.5)

Values are expressed as a median (interquartile range (IQR)) or *N* (%). AAV: ANCA-associated vasculitis; ANCA: antineutrophil cytoplasmic antibody; MPA: microscopic polyangiitis; EGPA: eosinophilic granulomatosis with polyangiitis; GPA: granulomatosis with polyangiitis; MPO: myeloperoxidase; P: perinuclear; PR3: proteinase 3; C: cytoplasmic; BVAS: Birmingham vasculitis activity score; FFS: five-factor score; ILD: interstitial lung disease; UIP: usual interstitial pneumonia.

**Table 2:** Comparison of ILD patterns among three AAV subtypes (*N* = 255).

Variables	MPA (*N* = 138)	EGPA (*N* = 52)	GPA (*N* = 65)	*P* value 1	*P* value 2	*P* value 3
ILD	42 (30.4)	3 (5.8)	8 (12.3)	<0.001	0.005	0.342
UIP	27/42 (64.3)	0/3 (0.0)	2/8 (25.0)	0.058	0.056	1.000
Non-UIP	15/42 (35.7)	3/3 (100.0)	6/8 (75.0)			

Values are expressed as a median *N* (%). *P* value 1: between MPA and EGPA groups. *P* value 2: between MPA and GPA groups. *P* value 3: between EGPA and GPA groups. ILD: interstitial lung disease; AAV: ANCA-associated vasculitis; ANCA: antineutrophil cytoplasmic antibody; MPA: microscopic polyangiitis; EGPA: eosinophilic granulomatosis with polyangiitis; GPA: granulomatosis with polyangiitis; UIP: usual interstitial pneumonia.

**Table 3:** Comparison of ILD patterns according to ANCA type (*N* = 255).

Variables	MPO-ANCA (or P-ANCA) negative (*N* = 85)	MPO-ANCA (or P-ANCA) positive (*N* = 170)	*P* value
ILD	5 (5.9)	48 (28.2)	<0.001
UIP	1 (1.2)	28 (16.5)	0.101
Variables	PR3-ANCA (or P-ANCA) negative (*N* = 211)	PR3-ANCA (or P-ANCA) positive (*N* = 44)	*P* value
ILD	51 (24.2)	2 (4.6)	0.004
UIP	29 (13.7)	0 (0)	0.113

Values are expressed as a median or *N* (%). ILD: interstitial lung disease; ANCA: antineutrophil cytoplasmic antibody; MPO: myeloperoxidase; P: perinuclear; PR3: proteinase 3; C: cytoplasmic; UIP: usual interstitial pneumonia.

**Table 4:** Comparison of pulmonary function tests according to ILD patterns (*N* = 45).

Variables	UIP (*N* = 26)	Non-UIP (*N* = 19)	*P* value
FVC (%)	82.0 (25.0)	85.0 (22.5)	0.621
DLCO (%)	74.0 (27.0)	68.0 (25.5)	0.818

Values are expressed as a median *N* (%). AAV: ANCA-associated vasculitis; ANCA: antineutrophil cytoplasmic antibody; ILD: interstitial lung disease; UIP: usual interstitial pneumonia; FVC: forced vital capacity; DLCO: diffusing capacity of the lung for carbon monoxide.

## Data Availability

The datasets used in the current study are available from the corresponding author on reasonable request.

## Supplementary Materials

Supplementary 1 Supplementary Table 1: comparison of ILD∗ patterns among three AAV subtypes in AAV patients (*N* = 255).

Supplementary 2 Supplementary Table 2: comparison of ILD∗ patterns according to ANCA type in AAV patients (*N* = 255).

Supplementary 3 Supplementary Table 3: comparison of pulmonary function tests according to ILD∗ patterns in AAV patients with ILD (*N* = 26).

Supplementary 4 Supplementary Fig. 1: comparison of cumulative patients' survival rates in AAV patients with ILD (*N* = 26). ILD: interstitial lung disease; UIP: usual pneumonia; AAV: ANCA-associated vasculitis; ANCA: antineutrophil cytoplasmic antibody.
