# Supplementary material for: Incidence and Patterns of Interstitial Lung Disease and Their Clinical Impact on Mortality in Patients with Antineutrophil Cytoplasmic Antibody-Associated Vasculitis: Korean Single-Centre Observational Study
Source: J Immunol Res. 2022 May 23;2022:2499404. doi: 10.1155/2022/2499404 (PMC9153384; doi:10.1155/2022/2499404)
Supplement: Supplementary 1 — Supplementary Table 1: comparison of ILD∗ patterns among three AAV subtypes in AAV patients (N = 255). [file 2499404.f1.docx]

**Supplementary Table 1. Comparison of ILD^*^ patterns among three AAV subtypes in AAV patients (N=255)**

| **Variables** | **MPA (N=138)** | **EGPA (N=52)** | **GPA (N=65)** | **P-value^1^** | **P-value^2^** | **P-value^3^** |
| --- | --- | --- | --- | --- | --- | --- |
| ILD | 16 (11.6) | 3 (5.8) | 7 (10.8) | 0.288 | 0.863 | 0.509 |
| UIP | 9/16 (56.3) | 0/16 (0) | 1/7 (14.3) | 0.211 | 0.089 | 1.000 |
| Non-UIP | 7/16 (43.7) | 3/16 (18.8) | 6/7 (85.7) |  |  |  |

Values are expressed as a median N (%).

^*^ILD: Only 26 patients, who had ILD after AAV diagnosis, were accepted as AAV patients with ILD, whereas, 27 patients, who had ILD at or before AAV diagnosis, were considered as AAV patients without ILD.

P-value^1^: between MPA and EGPA groups. P-value^2^: between MPA and GPA groups. P-value^3^: between EGPA and GPA groups.

ILD: interstitial lung disease; AAV: ANCA-associated vasculitis; ANCA: antineutrophil cytoplasmic antibody; MPA: microscopic polyangiitis; EGPA: eosinophilic granulomatosis with polyangiitis; GPA: granulomatosis with polyangiitis; UIP: usual interstitial pneumonia.
