# Supplementary material for: Incidence and Patterns of Interstitial Lung Disease and Their Clinical Impact on Mortality in Patients with Antineutrophil Cytoplasmic Antibody-Associated Vasculitis: Korean Single-Centre Observational Study
Source: J Immunol Res. 2022 May 23;2022:2499404. doi: 10.1155/2022/2499404 (PMC9153384; doi:10.1155/2022/2499404)
Supplement: Supplementary 3 — Supplementary Table 3: comparison of pulmonary function tests according to ILD∗ patterns in AAV patients with ILD (N = 26). [file 2499404.f3.docx]

**Supplementary Table 3. Comparison of pulmonary function tests according to ILD^*^ patterns in AAV patients with ILD (N=26)**

| **Variables** | **UIP**  **(N=10)** | **Non-UIP**  **(N=16)** | **p-value** |
| --- | --- | --- | --- |
| **FVC (%)** | 82.0 (23.5) | 85.0 (22.5) | 0.845 |
| **DLCO (%)** | 74.0 (15.0) | 70.0 (15.0) | 0.489 |

Values are expressed as a median N (%).

^*^ILD: Only 26 patients, who had ILD after AAV diagnosis, were accepted as AAV patients with ILD, whereas, 27 patients, who had ILD at or before AAV diagnosis, were considered as AAV patients without ILD.

AAV: ANCA-associated vasculitis; ANCA: antineutrophil cytoplasmic antibody; ILD: interstitial lung disease; UIP: usual interstitial pneumonia; FVC: forced vital capacity; DLCO: diffusing capacity of the lung for carbon monoxide.
