# Supplementary material for: Incidence and Patterns of Interstitial Lung Disease and Their Clinical Impact on Mortality in Patients with Antineutrophil Cytoplasmic Antibody-Associated Vasculitis: Korean Single-Centre Observational Study
Source: J Immunol Res. 2022 May 23;2022:2499404. doi: 10.1155/2022/2499404 (PMC9153384; doi:10.1155/2022/2499404)
Supplement: Supplementary 4 — Supplementary Fig. 1: comparison of cumulative patients' survival rates in AAV patients with ILD (N = 26). ILD: interstitial lung disease; UIP: usual pneumonia; AAV: ANCA-associated vasculitis; ANCA: antineutrophil cytoplasmic antibody. [file 2499404.f4.docx]

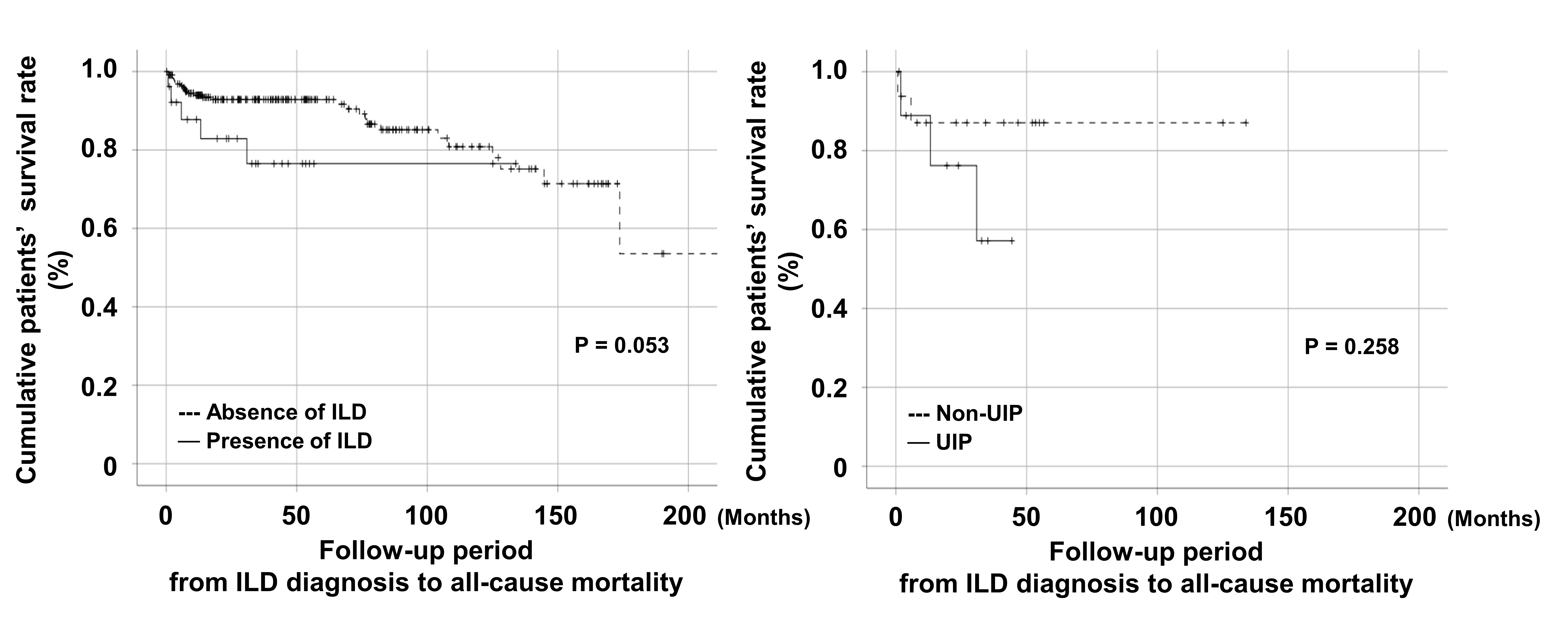


**Supplementary Fig. 1 Comparison of cumulative patients’ survival rates in AAV patients with** ILD (N=26).

ILD: interstitial lung disease; UIP: usual pneumonia; AAV: ANCA-associated vasculitis; ANCA: antineutrophil cytoplasmic antibody
